# Surgery of the Stomach

**Published:** 1905-09-16

**Authors:** 


### SURGERY OF THE STOMACH.

**Gastric Ulcer.**—The surgical treatment of gastric ulcer is one of great importance owing to the disastrous effects which result if the ulcer ruptures and permits the escape of intestinal contents into the general peritoneal cavity. A consideration of such cases suggests many points as referred to by Clarke.[1] What is the causation of these ulcers? Two main causes have been assigned for the formation of ulcers, namely thrombosis of the vessels of the stomach and the production of an excess of hydrochloric acid during the process of digestion. The difficulty of proof that either of these conditions is the chief factor in the causation of ulceration is, however, very great. One thing is absolutely certain, that whenever these cases have been under skilled observation before perforation has occurred, they are always found to be associated with indigestion, and usually with the presence of bad teeth. Their association with oral sepsis is undoubted, and the men in whom they occur, are generally addicted to drink. It is more than likely that no one cause is responsible for these ulcers. Of more importance to the surgeon is the position of these ulcers. It seems generally conceded that nearly all chronic ulcers, whether gastric or duodenal, occur in the neighbourhood of the pylorus; those that perforate are usually found near the lesser curvature, cardiac orifice, or on the back of the organ. Thus although Villard and Pinatelle,[2] in an analysis of 115 cases, found that gastric ulceration is very common about the lesser curvature, yet in only one-third of all cases of perforation is the ulcer situated in this region. This is to be accounted for by the high situation of the ulcer, its comparative fixity, its close apposition to other structures, and the frequent formation of adhesions, the latter tending to limit the extravasation and consequent inflammation, which often results in a perigastric abscess. Persistent vomiting is almost always a feature of perforation of an ulcer of the lesser curvature.

As Clarke[3] points out the symptoms of ruptured gastric ulcer vary considerably. It is very difficult usually to distinguish a gastric from a duodenal perforation before opening the abdomen, but the rapidity with which the symptoms come on after rupture is usually less after duodenal perforation than after gastric, probably because the duodenum contains less than the stomach, and also because duodenal perforations are, as a rule, smaller than gastric ones. The pain, too, is more often localised behind the right rectus in the case of a duodenal ulcer. When the abdomen has been opened for either of these two conditions, the diagnosis, as a rule, presents much less difficulty. The contents of the stomach consist of semi-digested food and are acid, while the contents of the duodenum, as a rule, are limpid, pale, and alkaline, and remind one almost cf the contents of an ovarian cyst. Duodenal contents, when they are extravasated almost invariably find their way down the right side of the mesentery into the right iliac fossa and pelvis, and thus it is that a duodenal perforation is more likely to be confounded with appendicitis than a gastric perforation is. Pain and shock are almost invariably present, but there are other conditions which give rise to both of these symptoms, and they must be excluded. The next point, therefore, is to determine the course and significance of these two symptoms. It will be found that a lack of abdominal movement is rarely absent in these cases. The abdominal movement is nearly always impaired in the region of the liver and stomach, and in ruptured duodenal ulcers more often on the right side of the abdomen than the left. Gentle palpation will reveal a painful area, possibly very limited in extent, and usually just behind one of the recti. The escape of free gas may also give rise to a diminution of the area of liver dulness, but too much reliance must not be placed on this symptom, as it may be simulated by intestine overlapping the liver. Clarke does not think it necessary to attempt to wash out every particle of food after the ulcer has been closed, but considers it a wise precaution to insert a drain for at least 24 hours in some part of the peritoneal cavity. Nothing should be given by the mouth for at least 24 or 48 hours after operation.

**Gastro enterostomy** in the treatment of gastric ulcer is very valuable both as an indirect means of stopping hæmorrhage and for the cure of chronic ulcers. Connell[4] gives many reasons why the direct methods (excision of ulcer, ligation of principal artery, cauterisation of ulcer, etc.) of dealing with hæmatemesis from ulceration are impracticable and frequently cannot be carried out. The difficulty of locating the ulcer is often very great, and the source of a hæmorrhage sufficient to cause death may easily escape detection. The fact that in 20 per cent. of the cases the lesion is multiple, the presence of firm and vascular adhesions and the indistinct limitations of the pathological tissues have all tended towards making impracticable the direct methods of attack of the bleeding point. This has been the cause of a general turning to the indirect methods, especially to that apparently all-healing operation, gastro-enterostomy. The exact manner in which a gastro-enterostomy usually causes a healing of the ulcer is generally explained in the following manner:—The anastomotic opening allows of a comparatively perfect drainage; the stomach is rapidly emptied and is therefore given rest and quiet; the hyperchlorhydria is diminished, and, as the opening is at the most dependent portion of the stomach, and to the left of the ulcer-bearing area, the ulcer is not irritated by prolonged contact with the stomach contents. The chief reason for expecting hæmorrhage to cease after the operation will be on account of the rest given to the stomach by the new opening, so, to a large extent doing away with peristalsis, and at the same time allowing the organ to contract; and also the lack of irritation of the bleeding-point by the stomach contents. These conditions and others do, without doubt, favour the stoppage of hæmorrhage and the formation of a clot. But that there are cases in which these measures are not sufficient to arrest the hæmorrhage cannot be doubted, and, therefore, as we are not able to determine with any reasonable degree of accuracy as to the nature of the source of the hæmorrhage, it would seem that a gastro-enterostomy is indicated only after a thorough search for the bleeding-point.

**Duodenal Ulcer** has, perhaps, not received the attention it deserves. It has been stated that all duodenal ulcers are secondary to gastric ulcers, but Mayo[5] says this has not been borne out by his experience. Considering the known errors and the possibilities springing from a pre-determined gastro-enterostomy and imperfect examination of an ulcer situated in the pyloric region, it must be evident that a duodenal ulcer is a far more common condition than has been thought. Mayo had 59 operations in 58 cases. Of these, seven were for acute conditions, developing, with one exception, upon chronic ulcer with three deaths. Fifty-one operations for chronic conditions gave one death. At the present time posterior gastro-enterostomy would appear to be the operation of choice in the chronic cases, but the last word has not yet been said. It is fairly certain that even with a large gastro-enterostomy the food will pass out by preference through an unobstructed pylorus by muscular action. Gastro-enterostomy performed for gastric ulcer is open to the same objection if there be no stenosis. For this reason, when the ulcer does not cause at least partial obstruction, it may be necessary to artificially block the pyloric outlet. After gastro-enterostomy the food, as a rule, passes out quickly, but there may be attacks of biliary regurgitation at intervals of days or weeks, and it is sometimes necessary to reoperate to check the disturbance. If the patient is in good condition Mayo now performs a posterior suture gastro-enterostomy after the clamp method introduced by Moynihan. Four inches below the completed gastro-enterostomy an entero-anastomosis, with suture between the two lines of the bowel, is made, using the holding clamps. To prevent bile arising to the level of the stomach and also to cause the food to always pass out by the efferent bowel, the afferent intestine between the entero-anastomosis and the gastro-enterostomy should be closed by an infolding stitch which turns the periphery of the intestine into the lumen or by other methods.

[1] Clin. Jour., Feb. 1, 1905, p. 241. [2] Med. Chron., Oct. 1904, p. 39. [3] Loc. cit. [4] Annals of Surg., Oct. 1904, p. 500. [5] Ibid., Dec. 1904, p. 886.

